# Clinical Correlates of Incidental Probable Benign Pulmonary Nodules with Diameters Less than 8 mm in a Healthy Korean Cohort: A Retrospective Study

**DOI:** 10.3390/jcm12237501

**Published:** 2023-12-04

**Authors:** Young Ju Jung, Hwajung Kim, Youngmee Kim, Won-Kyung Cho

**Affiliations:** 1 Department of Pulmonary and Critical Care Medicine, Health Screening and Promotion Center, Asan Medical Center, Seoul 05505, Republic of Korea; tazo76@naver.com; 2 Department of Clinical Epidemiology and Biostatistics, Asan Medical Center, Seoul 05505, Republic of Korea; hello.hello.hj@gmail.com; 3 Red Cross College of Nursing, Chung-Ang University, Seoul 06974, Republic of Korea; 4 Department of Pulmonary and Critical Care Medicine, International Healthcare Center, Asan Medical Center, University of Ulsan College of Medicine, Seoul 05505, Republic of Korea

**Keywords:** pulmonary nodules, benign, LDL cholesterol, cigarette smoking

## Abstract

Incidental pulmonary nodules detected via computed tomography (CT) are usually small, solid nodules (diameters less than 8 mm) that are likely benign and are difficult to biopsy. Additional features of the benignity of these small nodules may help determine the need and periodicity of further follow-up and should be identified. This study was conducted to examine the clinical factors associated with benign solid pulmonary nodules measuring less than 8 mm in diameter. This retrospective study enrolled participants who underwent low-dose chest CT scans for 3 consecutive years during routine health check-ups at a university hospital in Korea. We chose a 2-year study period to ensure that the nodule(s) were benign, which meant there was no interval change over this period. Participants were stratified into two groups: no nodule (*n* = 56) and nodule(s) (*n* = 355). Multivariable logistic regression analyses were performed to explore associations (adjusted odds ratio [aOR], 95% confidence interval [CI], *p*-value) between variables and nodule(s). In this study cohort, elevated levels of low-density lipoprotein (LDL) cholesterol were positively associated factors with the presence of benign pulmonary nodule(s) (aOR: 1.10, 95% CI:1.00–1.20, *p* = 0.0488), whereas current cigarette smoking was negatively associated with nodules (aOR: 0.26, 95% CI: 0.08–0.81, *p* = 0.0202). Therefore, an elevated LDL cholesterol level was the only factor that was positively associated with the presence of benign small pulmonary nodules.

## 1. Introduction

A pulmonary nodule is defined as a round intraparenchymal lung lesion measuring less than 3 cm in diameter [1]. The widespread availability of computed tomography (CT) and advances in CT technology have increased the incidental detection of pulmonary nodules.

Approximately 30% of all chest CT scans reveal at least one nodule [2], and the detection of a pulmonary nodule primarily generates concern about the probability of malignancy. Several guidelines recommend algorithms for pulmonary nodule management [3,4,5,6]; these recommendations are mainly based on the nodule density and size. With regard to nodule density, pulmonary nodules may be classified as solid, pure ground-glass, or partly solid nodules. Although solid nodules are most common, partly solid nodules have the highest potential for malignancy among pulmonary nodules of comparable sizes [7,8]. Based on their size, pulmonary nodules measuring less than 6 mm in diameter are correlated with a low cancer risk [4], whereas larger nodules (diameter greater than or equal to 8 mm) are associated with a higher risk for cancer [9].

Most pulmonary nodules measuring less than 8 mm in diameter are undetectable on chest X-rays and are only detected by CT. Therefore, pulmonary nodules that are incidentally detected by CT are mostly solid nodules that are less than 8 mm in diameter and are most likely to be benign [10,11]. These small nodules observed via CT are often interpreted by radiologists as ‘non-specific nodules’.

As pulmonary nodules that are less than 8 mm in diameter cannot be biopsied, these lesions necessitate follow-up if they are considered clinically important [7,9]. Identifying additional clinical features of the benignity of these small nodules will help determine the need for further follow-up.

In this study, the clinical characteristics of individuals with and without pulmonary benign nodules that are less than 8 mm in diameter were compared to identify clinical factors that are associated with the presence of these nodules.

## 2. Materials and Methods

### 2.1. Study Design, Data Sources, and Inclusion/Exclusion Criteria

We retrospectively screened 1738 participants who underwent three consecutive annual low-dose chest CT scans during routine health check-ups at the Health Screening and Promotion Center of the Asan Medical Center (Seoul, Republic of Korea) between January 2015 and December 2017. We used the 2-year time frame because a solid pulmonary nodule that remains stable for more than 2 years is likely benign [12]. These nodules are herein described as ‘benign’, yet a more precise term would be ‘probable benign nodules’ given the absence of histological verification.

For this study, the specific inclusion criteria for the nodule group (*n* = 355) were participants with stable non-calcified solid pulmonary nodule(s) that measured less than 8 mm in diameter, which could not be subjected to biopsy. In addition, the nodule(s) must have been observed with no interval change in three consecutive annual CT scans that were otherwise normal. When a participant had multiple pulmonary nodules, the participant was excluded if any of the pulmonary nodules had a diameter greater than 8 mm, ground-glass opacity, partly solid nature, or calcification. Multiple pulmonary nodules were defined as the detection of two or more nodules. Participants with three consecutive normal annual CT scans comprised the control (no-nodule) group (*n* = 56).

**Figure 1:**
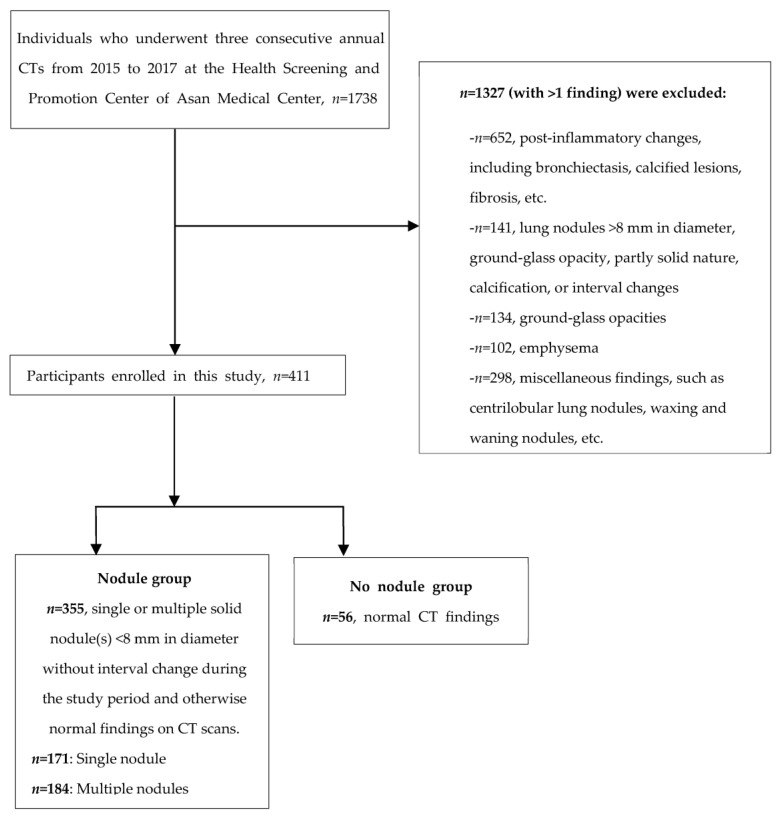
Flow diagram depicting the enrolment of participants in the study.

In this study cohort, 56 participants had normal CT findings, 171 had a single nodule measuring less than 8 mm in diameter, and 184 had multiple pulmonary nodules that were less than 8 mm in diameter (Figure 1).

### 2.2. CT Acquisition Parameters and Data Collection

Unenhanced low-dose chest CT scans were performed at a peak kilovoltage of 120 kV and a reference tube current of 50 mA using various multi-detector CT scanners as follows: two 64-detector row CT scanners (LightSpeed VCR, GE Medical Systems, Milwaukee, WI, USA) and a 128-detector row CT scanner (Discovery CT 750 HD, GE Medical Systems, Milwaukee, WI, USA). Patients were scanned craniocaudally from the apex of the lung to the costophrenic angle in the supine position at full inspiration during a single breath-hold. All CT images were reconstructed with 2.5 mm or thinner slices along the axial plane and a 3 mm slice in the coronal plane. Despite using two distinct scanners, nodule selection criteria were strictly confined to dimensions and density, minimizing the influence of varied CT environments on selection. Furthermore, with all readings subjected to independent review by radiologists via picture archiving and communication system software and consensus reached on the final determination, it is posited that the differences in CT environments exerted a negligible effect on target nodule selection.

A questionnaire was used to obtain detailed clinical information from the participants. The collected comorbidity data were confirmed using the medication history. For laboratory tests, blood was drawn early in the morning after overnight fasting. Esophagogastroduodenoscopy; breast, thyroid, or abdomen ultrasounds; or abdomen and pelvic CT scans were performed according to the patient’s preference. The presence of nodules, cysts, and polyps was considered a positive finding.

### 2.3. Statistical Analysis

Data analyses were conducted using SAS version 9.4 (SAS Institute, Inc., Cary, NC, USA). Student’s *t*-test, chi-square test, or Fisher’s exact test were conducted to evaluate the intergroup differences in continuous or categorical variables (Table 1, Table 2, Tables S1 and S2). Continuous variables are presented as mean ± standard deviation (SD), whereas categorical variables are presented as proportions (±SD).

Multiple logistic regression analyses were performed to identify the factors associated with the presence of benign pulmonary nodule(s). In the multiple logistic regression analyses, variables with *p* < 0.1 (Table 1 and Table 2), including sex, smoking status, blood glucose, and low-density lipoprotein (LDL) cholesterol level, were selected as potential predictors. The total cholesterol level as a variable was omitted from the multiple logistic regression because of multicollinearity between total cholesterol and LDL cholesterol (Table 3). Furthermore, multiple logistic regression analyses were performed to examine factors associated with multiple pulmonary nodules compared to a single nodule. Thus, variables with *p* < 0.1 (Tables S1 and S2), such as sex, weight, height, smoking status, creatinine, blood urea nitrogen (BUN), high-density lipoprotein (HDL) cholesterol, hemoglobin (Hb), *Helicobacter pylori* immunoglobulin G antibody, and free thyroxine (T4), were selected as potential predictors. Waist circumference (WC) was omitted from the multiple logistic regression due to multicollinearity between WC and body weight (Table S3). Moreover, we included drinking frequency in the multiple logistic analysis as some frequencies were significantly associated with nodules in the univariate logistic regression (Table S3), although this factor did not have a *p* < 0.1 (Table 1, Table 2, Tables S1 and S2).

The results are expressed using unadjusted and adjusted odds ratios (aORs) and 95% confidence intervals (CI). A *p*-value < 0.05 or a 95% CI that did not span 1.0 was considered statistically significant.

## 3. Results

### 3.1. Participants’ Sociodemographic Characteristics and Comorbidities

Of the 1738 individuals screened for inclusion in this study, 411 were enrolled (nodule group, *n* = 355; no nodule group, *n* = 56). Table 1 shows the sociodemographic characteristics and comorbidities of the participants stratified by the absence or presence of nodule(s). The participants’ mean ages were 53 and 52.5 years in the nodule and no nodule groups, respectively, and more men were included in both groups (nodule group: *n* = 47, 83.9%; no nodule group: *n* = 256, 72.1%). The proportion of current smokers was higher (*p* = 0.002) in the no nodule group (50.9%) than in the nodule group (27.9%). The mean body mass indices were less than 25 kg/m^2^ in both groups. Dyslipidemia and hypertension were the most common comorbidities in both groups.

### 3.2. Laboratory Test Results and Imaging Findings

Blood glucose levels were significantly lower in the nodule group than in the no nodule group (99.0 ± 16.8 vs. 105.2 ± 20.5, *p* = 0.035), whereas total cholesterol and LDL cholesterol levels were significantly higher in the nodule group than in the no nodule group (187.2 ± 37.1 vs. 175.1 ± 31.5, *p* = 0.021; 124.3 ± 34.1 vs. 112.7 ± 28.6, *p* = 0.016, respectively; Table 2). No other variables showed significant intergroup differences.

### 3.3. Factors Associated with Benign Pulmonary Nodule(s)

**Figure 2:**
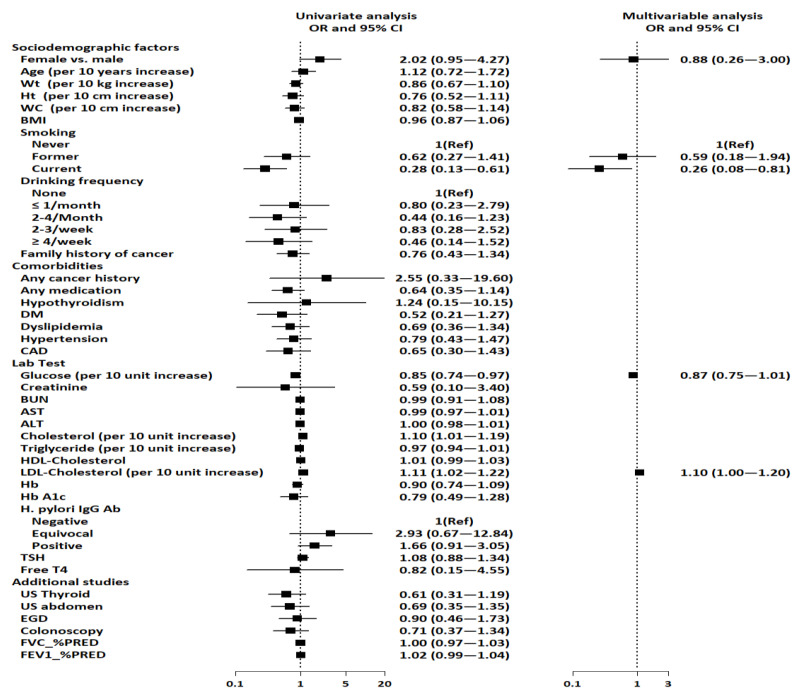
Forest plots depicting factors associated with benign pulmonary nodule(s). Abbreviations: ALT, alanine aminotransferase; AST, aspartate aminotransferase; BMI, body mass index; BUN, blood urea nitrogen; CAD, coronary artery disease; CI, confidence interval; DM, diabetes mellitus; EGD, esophagogastroduodenoscopy; FEV1, forced expiratory volume in one second; FVC, forced vital capacity; HDL, high-density lipoprotein; HbA1C, glycated hemoglobin; *H. pylori* IgG, *Helicobacter pylori* immunoglobulin G; LDL, low-density lipoprotein; PRED, predicted; REF, reference; T4, thyroxine; TSH, thyroid-stimulating hormone.

Table 3 and Figure 2 show the unadjusted and adjusted ORs for the factors associated with the presence of benign pulmonary nodule(s). Current smoking, lower glucose levels, and higher total and LDL cholesterol were associated with the presence of benign pulmonary nodule(s) after unadjusted logistic analysis. However, current smoking and LDL cholesterol were the only factors that were significantly associated with the presence of benign pulmonary nodule(s) after controlling for all relevant variables. This indicates that current smoking was negatively associated with the presence of benign pulmonary nodule(s) (aOR: 0.26, 95% CI: 0.08–0.81, *p* = 0.020). Furthermore, LDL cholesterol was associated with the presence of benign pulmonary nodule(s), indicating that participants with higher LDL cholesterol have a 1.10-fold higher risk for benign pulmonary nodule(s) (aOR: 1.10, 95% CI: 1.00–1.20, *p* = 0.049).

### 3.4. Single vs. Multiple Pulmonary Nodules

To examine the factors associated with multiplicity of nodules among the participants with pulmonary nodule(s), the sociodemographic characteristics, comorbidities, laboratory results, and imaging findings of the single and multiple nodule groups were compared (Tables S1 and S2). Weight, height, BUN, hemoglobin, and free T4 were significantly higher in the multiple pulmonary nodule group than in the single pulmonary nodule group. However, after adjusting for all variables, BUN was the only factor that was significantly associated with multiple pulmonary nodules (aOR: 1.09, 95% CI: 1.02–1.17, *p* = 0.016; Table S3 and Figure S1).

## 4. Discussion

Current guidelines for pulmonary nodule management are mainly based on nodule density and size [3,4,5,6], and they focus on ascertaining a probable malignant nature of the nodule. For solid nodules measuring 6 to 8 mm in diameter, follow-up with CT is recommended. However, further surveillance is generally not deemed necessary for nodules <6 mm, given that the associated cancer risk is <1% [7,9,13]. Nonetheless, there is a growing concern that such guidelines may not fully capture the malignancy risks of these small, solid lung nodules, which often elude detection on chest X-rays [7,9,14,15]. For instance, a study assessing the malignancy rate of small lung nodules via video-assisted thoracoscopic surgical biopsies in patients with no history of malignancy reported lung cancer in 14 out of 37 participants, indicating occurrences of either primary lung cancer or carcinoid tumors [14]. Furthermore, a retrospective study indicated a malignancy rate of 28% for nodules less than or equal to 4 mm in diameter detected by CT among 102 cancer patients [15]. While such findings suggest that the malignancy rates for these small, solid lung nodules may be underestimated by current guidelines, it remains imperative to balance the risk assessment with the need to minimize unnecessary CT follow-up.

Some additional radiologic features can also help differentiate between malignant and benign nodules. For instance, features that suggest benignity include a perifissural location, triangular morphology, or the presence of internal fat and benign calcifications. Malignancy is suspected in nodules presenting with spiculation, lobulation, pleural indentation, vascular convergence sign, bubble-like lucencies, or irregular air bronchogram [16].

In clinical practice, as previously noted, CT imaging is a cornerstone for the diagnosis and evaluation of pulmonary nodules. Nonetheless, there are inherent limitations to using CT as a solitary modality for distinguishing between benign nodules and malignancy, such as the occurrence of CT artifacts. Therefore, recognizing clinical characteristics that help differentiate nodules could, consequently, reduce the frequency of unnecessary CT scans. The clinical factors associated with lung cancer are extensively documented. These factors include cigarette smoking, age, family history, history of chronic lung disease, alcohol drinking, dietary factors, and environmental pollution [17]. However, clinical factors related to the benignity of nodules have not been systematically examined yet. With this background, we conducted this study to examine the clinical factors associated with benign small solid pulmonary nodules that are less than 8 mm in diameter and cannot be biopsied.

We found that an elevated LDL cholesterol level was the only factor that was positively associated with the presence of benign pulmonary nodules. To the best of our knowledge, no study has investigated the association of the blood lipid profile with benign pulmonary tumors. However, several studies have examined the relationship between the blood lipid profile and lung cancer. Intriguingly, most studies reported that low plasma HDL cholesterol was associated with an increased risk for lung cancer [18,19,20,21,22]. Furthermore, lower HDL cholesterol levels were related to lower survival rates in patients with non-small cell lung cancer [23]. It has been theorized that there is increased cholesterol synthesis in cancer cells in order to maintain the high rate of cell proliferation, and that HDL cholesterol may be a source of cholesterol for cancer cells by removing excess cholesterol from peripheral tissues [24]. In our study, HDL cholesterol was not significantly associated with the presence of benign nodules. Our novel finding of an association between elevated LDL cholesterol levels and the presence of small, benign pulmonary nodules presents an intriguing avenue for further inquiry. Given the paucity of research on this topic, our findings warrant additional verification. Research investigating the relationship between organ-specific nodules, such as those in the thyroid, and increased LDL cholesterol has yielded noteworthy associations, with several studies postulating potential mechanisms tied to obesity-related hormonal imbalances or insulin resistance [25,26]. Our results might suggest a broader paradigm wherein benign pulmonary nodules could be manifestations associated with metabolic dysregulation [25,26]. However, this hypothesis remains tentative and necessitates substantiation by further research. The incidence of dyslipidemia is known to increase with age; nevertheless, our data, as illustrated in Table 1, did not demonstrate a significant age discrepancy between participants with and without nodules. The age range was comparable across age groups, spanning 33 to 71 years in the ‘no nodule’ cohort and 38 to 78 years in the ‘nodule’ cohort, further substantiating the need for an age-independent exploration of this association.

Next, current cigarette smoking was negatively associated with the presence of benign pulmonary nodules. Cigarette smoking is a well-established risk factor for pulmonary cancer [27], which makes our findings quite intriguing. However, the interpretation of this finding is limited due to the design of this study that did not include participants with lung cancer. Overall, our study findings are in stark contrast with that of lung cancer, wherein the risk factors include smoking and low HDL cholesterol.

Participants in this study were selected based on their completion of three successive annual low-dose chest CT scans, a protocol grounded on the rationale that nodules stable for >2 years are likely benign [12]. The reasons for undergoing CT screening were not systematically recorded, reflecting the personal health management approach prevalent in Korea, where individuals bear the full cost and decision-making responsibility for their annual health assessments. Such autonomy extends to the selection of specific tests, typically informed by prior personal research.

Our study had a few limitations. First, we did not include data on any pathological diagnosis of these nodules. According to previous studies, the nodules described herein could include hamartoma, intrapulmonary lymph nodes, fibroma, hemangioma, leiomyoma, amyloidoma, pneumocytoma, granulomas, inflammatory nodules, or scars [28,29]. However, we focused on the factors associated with the general common features of small benign pulmonary nodules and not on factors related to each specific benign tumor. Second, we studied only non-biopsiable benign nodules that were less than 8 mm in diameter. Thus, uncertainty exists about whether our findings can be reproduced even in benign nodules that are greater than 8 mm in diameter.

Lastly, the notable association between high LDL cholesterol levels and the presence of benign pulmonary nodules determined in our study adds a fascinating dimension to the discourse on dyslipidemia as a metabolic disorder and its potential relationship with pulmonary nodule formation. The impact of this discovery is potentially limited, in part, by the comparative group selection—solely consisting of individuals with or without pulmonary nodules. The inclusion of a lung cancer group might have lent more weight to our findings. We initially intended to include such a group; however, a thorough examination of the data revealed a near absence of lung cancer patients, reflecting the generally healthy population that partakes in health screenings in Korea. This informed our decision to focus our classification and analysis on the binary presence or absence of benign nodules. The aim was to elucidate the clinical characteristics inherent in individuals with benign pulmonary nodules—an endeavor analogous to pinpointing susceptibility to mole development on the skin. Thus, even without a lung cancer group, the insights garnered from our research hold value. Yet, to affirm the generalizability of our findings, subsequent studies incorporating lung cancer patients are imperative.

## 5. Conclusions

We examined the clinical characteristics of probable benign solid pulmonary nodules that are less than 8 mm in diameter and found that an elevated LDL cholesterol level was the only factor that was positively associated with the presence of probable benign pulmonary nodules, whereas current smoking was associated with a lower incidence of probable benign pulmonary nodules. Although these findings are interesting, further research is required to fully put them into context. In particular, future research is needed to determine whether the results of our study are reproducible for probable benign pulmonary nodules when compared with malignant nodules.

## Figures and Tables

**Table 1:** Participants’ sociodemographics and comorbidities stratified by the absence or presence of lung nodules (*n* = 411).

Variables	No Nodule (*n* = 56)	Nodule(s) (*n* = 355)	*p*-Value
Sex, *n* (%)			0.062
Male	47 (83.9)	256 (72.1)	
Female	9 (16.1)	99 (27.9)	
Age (years), mean (SD)	52.5 (7.6)	53.0 (6.4)	0.616
Weight (kg), mean (SD)	71.7 (9.4)	69.6 (11.9)	0.150
Height (cm), mean (SD)	170.4 (6.6)	168.8 (8.0)	0.155
WC (cm), mean (SD)	87.8 (7.8)	86.4 (8.5)	0.232
BMI (kg/m^2^), mean (SD)	24.6 (2.7)	24.3 (2.9)	0.397
Smoking status, *n* (%)			0.002
Never	10 (18.9)	129 (36.3)	
Former	16 (30.2)	127 (35.8)	
Current	27 (50.9)	99 (27.9)	
Drinking frequency, *n* (%)			0.283
None	5 (9.4)	54 (15.2)	
≤1/month	6 (11.3)	52 (14.6)	
2–4/month	23 (43.4)	110 (31.0)	
2–3/week	11 (20.8)	99 (27.9)	
≥4/week	8 (15.1)	40 (11.3)	
Family history of cancer, *n* (%)	29 (52.7)	163 (45.9)	0.346
Taking any medicines, *n* (%)	34 (61.8)	180 (50.7)	0.125
Comorbidities, *n* (%)			
History of cancer	1 (1.8)	16 (4.5)	0.713
Hypothyroidism	1 (1.8)	8 (2.3)	1.000
DM	7 (12.7)	25 (7.0)	0.172
Dyslipidemia	14 (25.5)	68 (19.2)	0.277
Hypertension	17 (30.9)	93 (26.2)	0.463
CAD	9 (16.4)	40 (11.3)	0.278

Abbreviations: BMI, body mass index; CAD, coronary artery disease; DM, diabetes mellitus; SD, standard deviation; WC, waist circumference.

**Table 2:** Participants’ laboratory test results and imaging findings grouped by absence/presence of lung nodules (*n* = 411).

Variables	No Nodule (*n* = 56)	Nodule(s) (*n* = 355)	*p*-Value
Glucose (mg/dL), mean (SD)	105.2 (20.5)	99.0 (16.8)	0.035
Creatinine (mg/dL), mean (SD)	0.88 (0.18)	0.87 (0.16)	0.553
BUN (mg/dL), mean (SD)	13.5 (3.9)	13.4 (3.4)	0.856
AST (IU/L), mean (SD)	28.2 (13.8)	26.9 (11.0)	0.505
ALT (IU/L), mean (SD)	27.9 (12.7)	27.2 (16.7)	0.723
Total cholesterol (mg/dL), mean (SD)	175.1 (31.5)	187.2 (37.1)	0.021
Triglyceride (mg/dL), mean (SD)	138.7 (88.7)	121.2 (72.5)	0.165
HDL cholesterol (mg/dL), mean (SD)	52.0 (12.6)	54.5 (15.1)	0.230
LDL cholesterol (mg/dL), mean (SD)	112.7 (28.6)	124.3 (34.1)	0.016
Hemoglobin (g/dL), mean (SD)	15.0 (1.6)	14.8 (1.5)	0.287
HbA1C (%), mean (SD)	5.71 (0.69)	5.63 (0.52)	0.439
TSH (μU/mL), mean (SD)	2.26 (1.42)	2.42 (1.47)	0.448
Free T4 (ng/dL), mean (SD)	1.43 (0.19)	1.42 (0.16)	0.824
*H. pylori* IgG, *n* (%)			0.116
Negative	36 (64.3)	178 (50.1)	
Equivocal	2 (3.6)	29 (8.2)	
Positive	18 (32.1)	148 (41.7)	
Thyroid ultrasound, *n* (%)	74 (54.0)	96 (61.9)	0.171
Abdomen ultrasound, *n* (%)	127 (74.3)	127 (69.0)	0.274
EGD, *n* (%)	40 (24.5)	40 (23.3)	0.783
Colonoscopy, *n* (%)	83 (59.3)	93 (60.8)	0.794
FVC% predicted, mean (SD)	90.4 (9.2)	91.6 (9.7)	0.229
FEV1% predicted, mean (SD)	90.8 (10.0)	91.0 (9.9)	0.906

Abbreviations: ALT, alanine aminotransferase; AST, aspartate aminotransferase; BUN, blood urea nitrogen; EGD, esophagogastroduodenoscopy; FEV1, the first second of forced expiration; FVC, forced vital capacity; HDL, high-density lipoprotein; HbA1C, glycated hemoglobin; *H. pylori* IgG, *Helicobacter pylori* immunoglobulin G; LDL, low-density lipoprotein; T4, thyroxine; TSH, thyroid-stimulating hormone; SD, standard deviation.

**Table 3:** Results of the logistic regression analysis of risk factors for benign lung nodule(s) (*n* = 411).

	Univariate Logistic Regression		Multivariate Logistic Regression	
Variables	OR (95% CI)	*p*-Value	aOR (95% CI)	*p*-Value
Female sex	2.02 (0.95–4.27)	0.066	0.88 (0.26–3.00)	0.844
Age (per 10-year increase)	1.12 (0.72–1.72)	0.615		
Weight (per 10 kg increase)	0.86 (0.67–1.10)	0.221		
Height (per 10 cm increase)	0.76 (0.52–1.11)	0.156		
WC (per 10 cm increase)	0.82 (0.58–1.14)	0.231		
BMI	0.96 (0.87–1.06)	0.397		
Smoking status				
Never	REF		REF	
Former	0.62 (0.27–1.41)	0.250	0.59 (0.18–1.94)	0.388
Current	0.28 (0.13–0.61)	0.001	0.26 (0.08–0.81)	0.020
Drinking frequency				
None	REF			
≤1/month	0.80 (0.23–2.79)	0.729		
2–4/month	0.44 (0.16–1.23)	0.118		
2–3/week	0.83 (0.28–2.52)	0.747		
≥4/week	0.46 (0.14–1.52)	0.205		
Family history of cancer	0.76 (0.43–1.34)	0.347		
Comorbidities				
History of any cancer	2.55 (0.33–19.60)	0.369		
Any medication use	0.64 (0.35–1.14)	0.127		
Hypothyroidism	1.24 (0.15–10.15)	0.838		
DM	0.52 (0.21–1.27)	0.150		
Dyslipidemia	0.69 (0.36–1.34)	0.279		
Hypertension	0.79 (0.43–1.47)	0.464		
CAD	0.65 (0.30–1.43)	0.281		
Glucose (per 10 mg/dL increase)	0.85 (0.74–0.97)	0.017	0.87 (0.75–1.01)	0.072
Creatinine	0.59 (0.10–3.40)	0.552		
BUN	0.99 (0.91–1.08)	0.856		
AST	0.99 (0.97–1.01)	0.429		
ALT	1.00 (0.98–1.01)	0.770		
Total cholesterol (per 10 mg/dL increase)	1.10 (1.01–1.19)	0.021		
Triglyceride (per 10 mg/dL increase)	0.97 (0.94–1.01)	0.110		
HDL cholesterol	1.01 (0.99–1.03)	0.230		
LDL cholesterol (per 10 mg/dL increase)	1.11 (1.02–1.22)	0.017	1.10 (1.00–1.20)	0.049
Hemoglobin	0.90 (0.74–1.09)	0.287		
HbA1C	0.79 (0.49–1.28)	0.337		
*H. pylori* IgG				
Negative	REF			
Equivocal	2.93 (0.67–12.84)	0.153		
Positive	1.66 (0.91–3.05)	0.100		
TSH	1.08 (0.88–1.34)	0.447		
Free T4	0.82 (0.15–4.55)	0.824		
Thyroid ultrasound	0.61 (0.31–1.19)	0.148		
Abdomen ultrasound	0.69 (0.35–1.35)	0.277		
EGD	0.90 (0.46–1.73)	0.745		
Colonoscopy	0.71 (0.37–1.34)	0.289		
FVC% PRED (per 1% PRED increase)	1.00 (0.97–1.03)	0.945		
FEV1% PRED (per 1% PRED increase)	1.02 (0.99–1.04)	0.262		

Abbreviations: ALT, alanine aminotransferase; aOR, adjusted odds ratio; AST, aspartate aminotransferase; BMI, body mass index; BUN, blood urea nitrogen; CAD, coronary artery disease; CI, confidence interval; DM, diabetes mellitus; EGD, esophagogastroduodenoscopy; FEV1, the first second of forced expiration; FVC, forced vital capacity; HDL, high-density lipoprotein; HbA1C, glycated hemoglobin; *H. pylori* IgG, *Helicobacter pylori* immunoglobulin G; LDL, low-density lipoprotein; PRED, predicted; REF, reference; T4, thyroxine; TSH, thyroid-stimulating hormone.

## Data Availability

All data generated or analyzed during this study are included in this published article.

## Supplementary Materials

The following supporting information can be downloaded at: https://www.mdpi.com/article/10.3390/jcm12237501/s1, Figure S1: Forest plots showing the factors associated with multiple lung nodules; Table S1: Participants’ sociodemographics and comorbidities in subgroups with single or multiple nodules (*n* = 355); Table S2: Participants’ laboratory test results and imaging findings by subgroups with single or multiple nodules (*n* = 355); Table S3: Results of logistic regression of risk factors for multiple (single vs. multiple) lung nodules (*n* = 355).
