# Supplementary material for: Clinical Correlates of Incidental Probable Benign Pulmonary Nodules with Diameters Less than 8 mm in a Healthy Korean Cohort: A Retrospective Study
Source: J Clin Med. 2023 Dec 4;12(23):7501. doi: 10.3390/jcm12237501 (PMC10707178; doi:10.3390/jcm12237501)
Supplement: Supplementary file 1 [file jcm-12-07501-s001.zip › jcm-2680065-supplementary.pdf]

Journal of
Clinical Medicine

Table S1. Participants' sociodemographics and comorbidities in subgroups with single or multiple nodules (N=355).

Variables	Single nodule (n = 171)	Multiple nodules (n =184)	*p*-value
Sex, n (%)			
Male	113 (66.1)	143 (77.7)	0.015
Female	58 (33.9)	41 (22.3)	
Age (years), mean (SD)	52.6 (6.4)	53.4 (6.4)	0.248
Weight (kg), mean (SD)	68.2 (12.3)	70.9 (11.3)	0.034
Height (cm), mean (SD)	167.8 (8.3)	169.7 (7.6)	0.024
WC (cm), mean (SD)	85.5 (8.7)	87.2 (8.3)	0.059
BMI (kg/m^2), mean (SD)	24.1 (3.1)	24.5 (2.7)	0.181
Smoking, n (%)			0.058
Never	72 (42.1)	57 (31.0)	
Former	52 (30.4)	75 (40.8)	
Current	47 (27.5)	52 (28.3)	
Drinking frequency, n (%)			0.319
None	33 (19.3)	21 (11.4)	
≤1/month	25 (14.6)	27 (14.7)	
2–4/month	48 (28.1)	62 (33.7)	
2–3/week	47 (27.5)	52 (28.3)	
≥4/week	18 (10.5)	22 (12.0)	
Family history of cancer, n (%)	74 (43.3)	89 (48.4)	0.336
Taking any medicines, n (%)	82 (48.0)	98 (53.3)	0.318
Comorbidities, n (%)			
History of any cancer	8 (4.7)	8 (4.3)	0.881
Hypothyroidism	4 (2.3)	4 (2.2)	1.000
DM	12 (7.0)	13 (7.1)	0.986
Dyslipidaemia	33 (19.3)	35 (19.0)	0.947
Hypertension	42 (24.6)	51 (27.7)	0.499
CAD	18 (10.5)	22 (12.0)	0.670

Abbreviations: BMI, body mass index; CAD, coronary artery disease; DM, diabetes mellitus; SD, standard deviation; WC, waist circumference.

Table S2. Participants' laboratory test results and imaging findings by subgroups with single or multiple nodules (N=355).

Variables	Single nodule (n = 171)	Multiple nodules (n = 184)	*p*-value
Glucose (mg/dL), mean (SD)	99.5 (16.4)	98.4 (17.2)	0.548
Creatinine (mg/dL), mean (SD)	0.85 (0.17)	0.88 (0.14)	0.072
BUN (mg/dL), mean (SD)	12.9 (3.0)	13.9 (3.6)	0.0050
AST (IU/L), mean (SD)	26.4 (10.4)	27.3 (11.5)	0.442
ALT (IU/L), mean (SD)	26.7 (17.5)	27.7 (16.0)	0.551
Total cholesterol (mg/dL), mean (SD)	186.6 (37.8)	187.8 (36.4)	0.754
Triglyceride, mean (SD)	115.8 (62.6)	126.2 (80.5)	0.173
HDL cholesterol (mg/dL), mean (SD)	56.1 (15.7)	53.1 (14.5)	0.065
LDL cholesterol (mg/dL), mean (SD)	123.1 (35.3)	125.4 (33.1)	0.534
Haemoglobin (g/dL), mean (SD)	14.6 (1.6)	14.9 (1.4)	0.043
HbA1C (%), mean (SD)	5.62 (0.50)	5.64 (0.54)	0.746
TSH (μU/mL), mean (SD)	2.42 (1.37)	2.42 (1.56)	1.000
Free T4 (ng/dL), mean (SD)	1.40 (0.16)	1.44 (0.16)	0.031
H. pylori IgG Ab, n (%)			0.022
Negative	89 (52.0)	89 (48.4)	
Equivocal	20 (11.7)	9 (4.9)	

Positive	62 (36.3)	86 (46.7)	
Thyroid ultrasound, n (%)	74 (54.0)	96 (61.9)	0.171
Abdomen ultrasound, n (%)	127 (74.3)	127 (69.0)	0.274
EGD, n (%)	40 (24.5)	40 (23.3)	0.783
Colonoscopy, n (%)	83 (59.3)	93 (60.8)	0.794
FVC % predicted, mean (SD)	90.4 (9.2)	91.6 (9.7)	0.229
FEV1 % predicted, mean (SD)	90.8 (10.0)	91.0 (9.9)	0.905

Abbreviations: ALT, alanine aminotransferase; AST, aspartate aminotransferase; BUN, blood urea nitrogen; EGD, esophagogastroduodenoscopy; FEV1, the first second of forced expiration; FVC, forced vital capacity; HDL, high-density lipoprotein; HbA1C, glycated haemoglobin; H. pylori IgG, Helicobacter pylori immunoglobulin G; LDL, low-density lipoprotein; T4, thyroxine; TSH, thyroid-stimulating hormone; SD, standard deviation.

Table S3. Results of logistic regression of risk factors for multiple (single vs. multiple) lung nodules (N=355).

	**Univariate logistic regression**		**Multivariate logistic regression**	
**Variables**	**OR (95% CI)**	***p*-value**	**aOR (95% CI)**	***p*-value**
Female, sex	0.56 (0.35–0.89)	0.015		
Age (per 10-year increase)	1.21 (0.87–1.69)	0.248	0.96 (0.31–3.01)	0.944
Weight (per 10-kg increase)	1.21 (1.01–1.45)	0.035		
Height (per 10-cm increase)	1.36 (1.04–1.77)	0.025	1.02 (0.75–1.39)	0.883
WC (per 10-cm increase)	1.27 (0.99–1.63)	0.060	1.01 (0.60–1.69)	0.974
BMI	1.05 (0.98–1.13)	0.179		
Smoking				
Never	REF		REF	
Former	1.82 (1.11–2.99)	0.018	1.38 (0.63–3.01)	0.416
Current	1.40 (0.83–2.36)	0.212	1.15 (0.52–2.55)	0.731
Drinking frequency				
None	REF		REF	
≤1/month	1.70 (0.78–3.67)	0.179	1.79 (0.79–4.03)	0.161
2–4/month	2.03 (1.04–3.94)	0.037	1.75 (0.81–3.75)	0.152
2–3/week	1.74 (0.89–3.41)	0.108	1.37 (0.60–3.10)	0.451
≥4/week	1.92 (0.84–4.40)	0.123	1.51 (0.57–4.00)	0.402
Family history of cancer	1.23 (0.81–1.87)	0.336		
Comorbidities				
History of any cancer	0.93 (0.34–2.52)	0.881		
Any medication	1.24 (0.82–1.88)	0.318		
Hypothyroidism	0.93 (0.23–3.77)	0.917		
DM	1.01 (0.45–2.27)	0.986		
Dyslipidaemia	0.98 (0.58–1.67)	0.948		
Hypertension	1.18 (0.73–1.89)	0.499		
CAD	1.15 (0.60–2.23)	0.671		
Glucose (per 10-unit increase)	0.96 (0.85–1.09)	0.548		
Creatinine	3.47 (0.90–13.39)	0.071	0.57 (0.08–4.09)	0.572
BUN	1.09 (1.03–1.17)	0.006	**1.09 (1.02–1.17)**	**0.016**
AST	1.01 (0.99–1.03)	0.442		
ALT	1.00 (0.99–1.02)	0.550		
Total cholesterol (per 10-unit increase)	1.01 (0.95–1.07)	0.753		
Triglyceride (per 10-unit increase)	1.02 (0.99–1.05)	0.181		
HDL cholesterol	0.99 (0.97–1.00)	0.064	0.99 (0.98–1.01)	0.507
LDL cholesterol (per 10-unit increase)	1.02 (0.96–1.08)	0.533		
Haemoglobin	1.16 (1.00–1.34)	0.044	1.08 (0.87–1.34)	0.486
HbA1C	1.07 (0.71–1.60)	0.746		

H. pylori IgG Ab				
Negative	REF		REF	
Equivocal	0.45 (0.19–1.04)	0.063	0.44 (0.18–1.04)	0.062
Positive	1.39 (0.89–2.15)	0.144	1.44 (0.91–2.29)	0.119
TSH	1.00 (0.87–1.15)	1.000		
Free T4	4.28 (1.13–16.20)	0.032	3.78 (0.92–15.52)	0.065
Thyroid ultrasound	1.39 (0.87–2.21)	0.171		
Abdomen ultrasound	0.77 (0.49–1.23)	0.274		
Esophagogastroduodenoscopy	0.93 (0.56–1.54)	0.783		
Colonoscopy	1.06 (0.67–1.70)	0.794		
FVC % PRED (per 1% PRED increase)	1.01 (0.99–1.04)	0.228		
FEV1 % PRED (per 1% PRED increase)	1.00 (0.98–1.02)	0.905		

Abbreviations: ALT, alanine aminotransferase; aOR, adjusted odds ratio; AST, aspartate aminotransferase; BMI, body mass index, BUN, blood urea nitrogen; CAD, coronary artery disease; DM, diabetes; FEV1, the first second of forced expiration; FVC, forced vital capacity; HDL, high-density lipoprotein; HbA1C, glycated haemoglobin; H. pylori IgG, Helicobacter pylori immunoglobulin G; LDL, low-density lipoprotein; PRED, predicted; REF, reference; T4, thyroxine; TSH, thyroid-stimulating hormone.

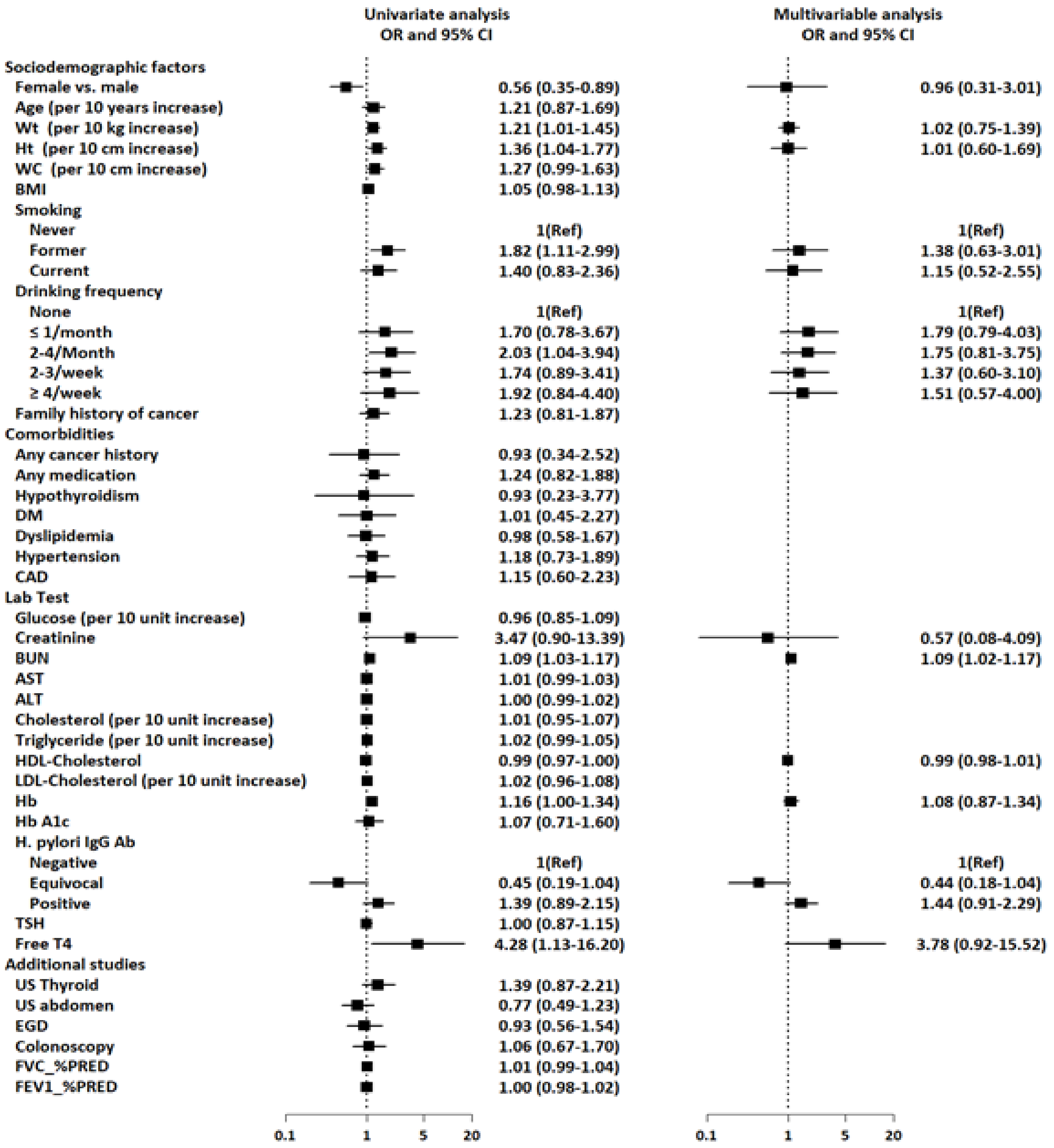

Figure S1. Forest plots showing the factors associated with multiple lung nodules. Abbreviations**:** ALT, alanine aminotransferase; AST, aspartate aminotransferase; BMI, body mass index, BUN, blood urea nitrogen; CAD, coronary artery disease; CI, confidence interval; DM, diabetes mellitus; EGD, esophagogastroduodenoscopy; FEV1, the first second of forced expiration; FVC, forced vital capacity; HDL, high-density lipoprotein; HbA1C, glycated haemoglobin; *H. pylori* IgG, *Helicobacter pylori* immunoglobulin G; LDL, low-density lipoprotein; PRED, predicted; REF, reference; T4, thyroxine; TSH, thyroid-stimulating hormone.
